# Prediction Models for Prognosis of Cervical Cancer: Systematic Review and Critical Appraisal

**DOI:** 10.3389/fpubh.2021.654454

**Published:** 2021-05-07

**Authors:** Bingjie He, Weiye Chen, Lili Liu, Zheng Hou, Haiyan Zhu, Haozhe Cheng, Yixi Zhang, Siyan Zhan, Shengfeng Wang

**Affiliations:** ^1^Department of Epidemiology and Biostatistics, School of Public Health, Peking University Health Science Center, Beijing, China; ^2^Department of Obsterics and Gynecology, Peking University Third Hospital, Beijing, China; ^3^School of Public Health, Peking University Health Science Center, Beijing, China

**Keywords:** cervical cancer, prediction model, predictors, risk of bias, statistical analysis

## Abstract

**Objective:** This work aims to systematically identify, describe, and appraise all prognostic models for cervical cancer and provide a reference for clinical practice and future research.

**Methods:** We systematically searched PubMed, EMBASE, and Cochrane library databases up to December 2020 and included studies developing, validating, or updating a prognostic model for cervical cancer. Two reviewers extracted information based on the CHecklist for critical Appraisal and data extraction for systematic Reviews of prediction Modeling Studies checklist and assessed the risk of bias using the Prediction model Risk Of Bias ASsessment Tool.

**Results:** Fifty-six eligible articles were identified, describing the development of 77 prognostic models and 27 external validation efforts. The 77 prognostic models focused on three types of cervical cancer patients at different stages, i.e., patients with early-stage cervical cancer (*n* = 29; 38%), patients with locally advanced cervical cancer (*n* = 27; 35%), and all-stage cervical cancer patients (*n* = 21; 27%). Among the 77 models, the most frequently used predictors were lymph node status (*n* = 57; 74%), the International Federation of Gynecology and Obstetrics stage (*n* = 42; 55%), histological types (*n* = 38; 49%), and tumor size (*n* = 37; 48%). The number of models that applied internal validation, presented a full equation, and assessed model calibration was 52 (68%), 16 (21%), and 45 (58%), respectively. Twenty-four models were externally validated, among which three were validated twice. None of the models were assessed with an overall low risk of bias. The Prediction Model of Failure in Locally Advanced Cervical Cancer model was externally validated twice, with acceptable performance, and seemed to be the most reliable.

**Conclusions:** Methodological details including internal validation, sample size, and handling of missing data need to be emphasized on, and external validation is needed to facilitate the application and generalization of models for cervical cancer.

## Introduction

Cervical cancer is one of the leading causes of cancer-related death among women and ranks fourth among the most common malignancy in women in 2020 worldwide (1, 2). In 2020, there were an estimated 604,127 new cases and more than 341,831 deaths from cervical cancer, representing 7.7% of all female cancer deaths around the world (2). Cervical cancer patients have a poor prognosis in developing countries (3, 4). Improvement in survival remains the ultimate goal of treatment in the clinical setting, and treatment varies according to the stage, metastasis, or recurrence (5, 6). The physician can use known factors to check the personal prognosis and predict the clinical outcomes after a specific therapy such as radical hysterectomy or radiotherapy (7, 8). The prediction of potential personal prognosis facilitates the clinicians to decide the subsequent therapies or follow-up examinations (9, 10). For example, an American study indicated that clinicians could get a net benefit of 0.35 (the benefit from treating true positive patients minus the harm from treating those who do not need treatment) with only delivering intensive therapy to patients with a high risk of 3-year survival (threshold probability: about 0.76) based on the prognostic model, compared with delivering therapy to all patients (11).

A prognostic model, combining a few known factors to predict the individual risk of occurrence, progression, and clinical outcome of cervical cancer, is critical in practice (12). A couple of models have shown great positive net benefits across wide ranges of risk, indicating their favorable clinical utility (11, 13, 14). A prognostic model is a formal combination, usually a statistical equation, of multiple predictors, from which risks of a specific outcome can be calculated for individuals (15–17). As for cervical cancer patients, due to the limited predictive value for the classification of the International Federation of Gynecology and Obstetrics (FIGO) alone, a couple of prognostic models have been proposed to predict and guide treatments based on different tumor and demographic characteristics (13, 18). However, the uneven quality and the diversity of the clinical settings, outcomes, and predictors may limit the practicality of models, and systematic reviews on prognostic models of other diseases also suggested that the methodological features of existing studies varied (19–21). No comprehensive evaluation of prognostic models for cervical cancer has been done. Therefore, in order to help clinicians and the public to select the most appropriate models in practice, there is an urgent need to systematically summarize these models, map their characteristics, and examine their performance.

In this study, we conducted a systematic review to identify and summarize these prognostic models for cervical cancer patients and assess their qualities based on the guideline of the CHecklist for critical Appraisal and data extraction for systematic Reviews of prediction Modeling Studies (CHARMS) and the Prediction model Risk Of Bias ASsessment Tool (PROBAST) (22, 23) in order to provide evidence for determining reliable models and to provide a methodology reference for future research within this field.

## Materials and Methods

The Preferred Reporting Items for Systematic Reviews and Meta-analyses (PRISMA) guidelines guided this systematic review. Supplementary Material 1 shows the PRISMA checklist.

### Information Sources and Search Strategy

We systematically searched PubMed, EMBASE, and Cochrane library databases from their inception to December 31, 2020 for relevant articles. We combined the following search terms which were used in referring to prediction models (predict^*^, progn^*^, risk score, risk calculation, risk assessment, c statistic, discrimination, calibration, AUC, area under the curve, area under the receiver operator characteristic curve) and the disease (cervical, cervix; cancer^*^, carcinoma^*^, neoplasm^*^, and tumor^*^) based on previous research (19, 24) (^*^ means asterisk wildcard). No other filters were applied. Details of the search strategy are given in Supplementary Material 2.

### Eligibility Criteria

Studies were included if they reported the development, the update, or the external validation of at least one multivariable prognostic model based on individual characteristics, and the outcome of the prognostic model was any clinical outcome (recurrence, metastasis, death, *etc*.) in patients diagnosed with cervical cancer (Table 1). Only published studies were considered.

**Table 1:** Key items for framing the aim, search strategy, and study inclusion and exclusion criteria for systematic review.

**Item**	**Definition**
Population	Patients diagnosed as having cervical cancer
Intervention	Any prognostic model to predict clinical outcomes (recurrence, metastasis, death, *etc*.) in cervical cancer patients
Comparator	Not applicable
Outcomes	Any outcome reported by prognostic models
Timing	No restriction
Setting	No restriction

Studies were not eligible for inclusion if they (1) were conference abstracts, letters, and editorials or non-original studies such as reviews, (2) only examined independent prognostic factors, (3) focused on methodology, (4) developed or validated diagnostic or screening models, (5) did not construct a model to estimate individual risks, and (6) did not have a full text in English or available full text.

### Study Selection

Study selection was conducted using EndNote X9. Duplicates were found and removed automatically and manually. Preliminary screening was performed through reviewing of the titles and abstracts by two independent reviewers with backgrounds in obstetrics and gynecology and uniform training, using pre-defined eligibility criteria. The full texts of the included articles after preliminary screening were reviewed independently by two reviewers. Any disagreement between reviewers was resolved by consensus.

### Data Extraction

For each relevant publication, two reviewers extracted information through a piloted standardized form based on the recommendations in the CHARMS checklist (22). The key items to be extracted from each primary study were grouped within 11 domains, including source of data, participants, outcome(s) to be predicted, candidate predictors, sample size, missing data, model development, model performance, model evaluation, results, interpretation, and discussion. In addition, we extracted the general characteristics of the studies, including title, author, publication year, and specific objective (i.e., to develop or to validate or both). Model performance is typically evaluated using measures of calibration and discrimination. Calibration reflects the disparity between predictions and observed outcomes. Discrimination reflects the ability of a prognostic model to distinguish between individuals who do or do not develop the outcome (25, 26). Discrimination metrics are usually represented by concordance index (C-index) or area under the receiver operator characteristic curve (AUROC). A discrimination metric of 0.5 describes a random prediction, whereas a perfectly predicting model would have a discrimination metric of 1.0. The methods used to assess model calibration and discrimination should be suitable for the corresponding model (25).

The included models can be divided into three categories based on the FIGO stage of the research population: models for patients with early-stage cervical cancer, models for patients with locally advanced cervical cancer, and models for all-stage (including early stage and locally advanced) cervical cancer patients.

### Assessment of Risk of Bias

The PROBAST was used to assess the risk of bias (ROB) of each prognostic model identified from the included studies (23, 25). Two investigators assessed ROB for each model independently. Applicability is beyond the scope of this study due to the lack of specific questions in the population, predictors, or outcome. The assessment of ROB in PROBAST involved four domains (participants, predictors, outcome, and analysis), and 20 signaling questions covered the key aspects of prediction model studies. Each question was answered as “yes,” “probably yes,” “probably no,” “no,” or “no information” according to the indications provided by PROBAST, and the ROB of each domain was judged as low, high, or unclear based on the questions in the corresponding domains. The overall assessment of each model was classified as low or high ROB. When all domains were judged as low risk, the overall risk was classified as low ROB; as long as one domain was considered high risk, the overall risk was classified as high ROB.

### Data Synthesis

We conducted a descriptive analysis of the characteristics of models and reported mean or median for continuous variables, with differences calculated using *t*-test or Kruskal–Wallis test, or percentages for categorical variables, with differences calculated using χ^2^ test or Fisher's exact test. We compared the number of sample size and events and the proportion of methodological items and predictors used in the models across three categories. We used Stata15.0 for the statistical analysis.

## Results

**Figure 1:**
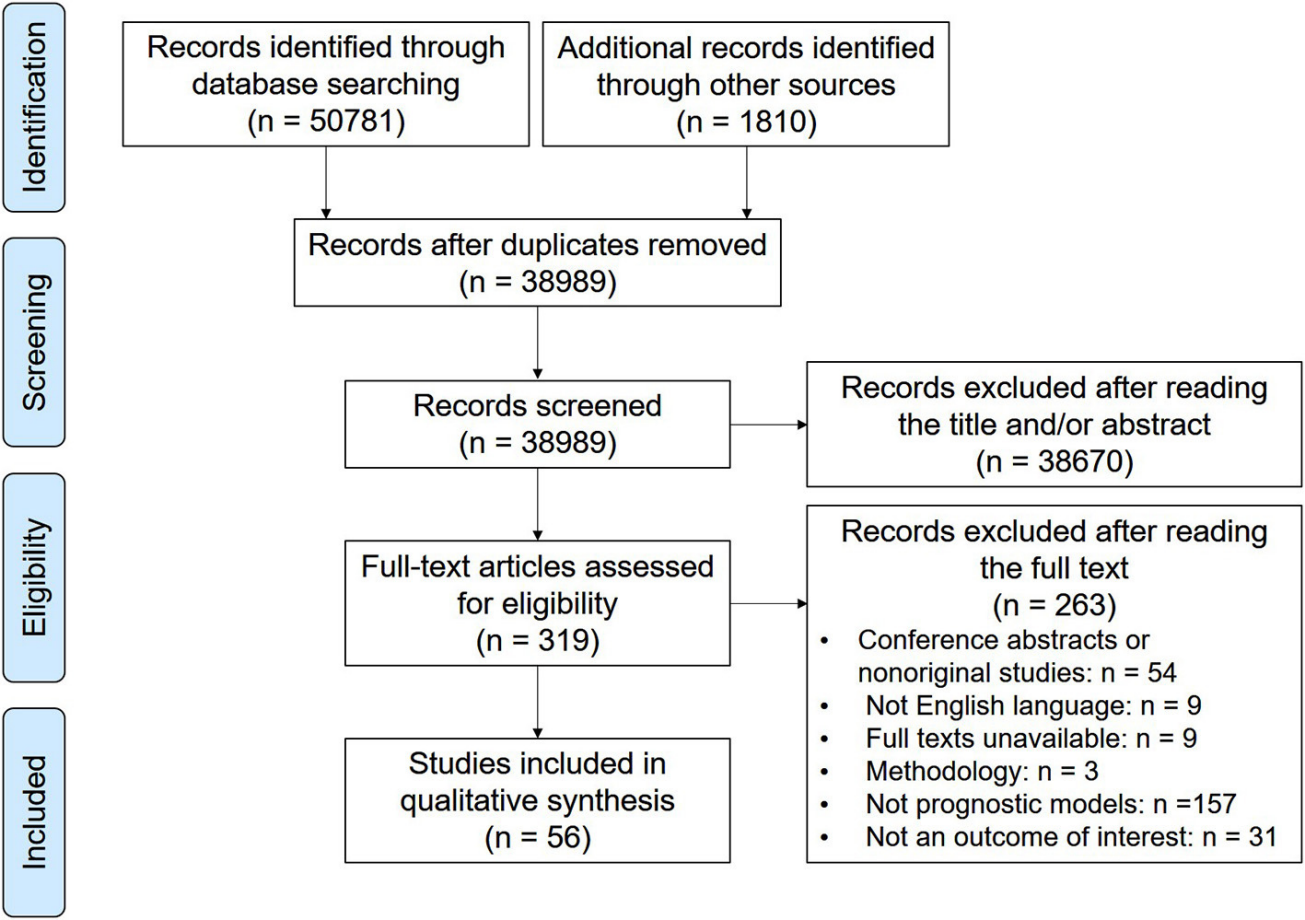
Flow chart of the literature search for prognostic models related to cervical cancer.

Of the 52,591 screened articles, 56 articles describing the development of 77 prognostic models and the external validation of 24 prognostic models for cervical cancer patients were eligible (Figure 1). The number of prognostic models increased steadily in the past decade, tripling in annual growth in 2019. The prognostic models were mainly developed in the United States (*n* = 25; 32%), China (*n* = 23; 30%), and Korea (*n* = 18; 23%). External validation was performed only in Korea (*n* = 10; 37%), Spain (*n* = 8; 30%), China (*n* = 8; 30%), and the United States (*n* = 1; 4%). For the derivation cohorts, the median sample size and the median number of events were 549 (interquartile range, 203.5–843) and 77 (40.5–187), respectively. For the internal validation cohorts, the median sample size and the median number of events were 484.5 (234–833) and 95 (46–187), respectively.

**Figure 2:**
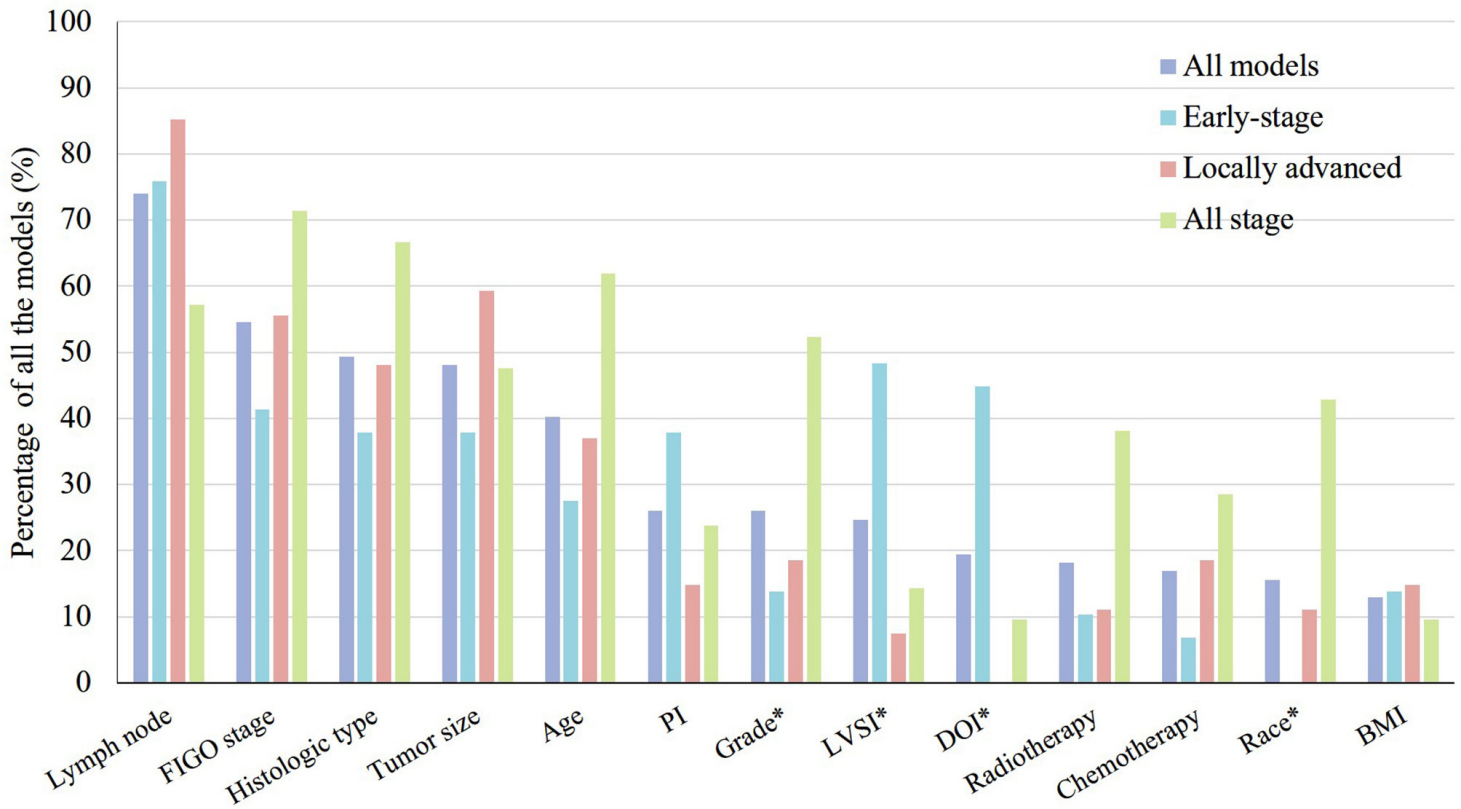
Thirteen most frequently used predictors in 77 prognostic models for the prognosis of cervical cancer patients presented by clinical stage. PI, parametrium invasion; LVSI, lymph vascular space invasion; DOI, depth of stromal invasion; BMI, body mass index. *p < 0.05.

The research population of eligible models covered all stages of cervical cancer, including patients with early-stage cervical cancer (*n* = 29; 38%), patients with locally advanced cervical cancer (*n* = 27; 35%), and all-stage cervical cancer patients (*n* = 21; 27%) (Supplementary Material 3). These models also focused on a wide range of clinical outcomes. The most common clinical outcomes were overall survival (*n* = 34; 44%), disease-free survival (*n* = 12; 16%), progression-free survival (*n* = 9; 12%), and disease-specific survival (*n* = 7; 9%). The predictors encountered most frequently were lymph nodes status (*n* = 57; 74%), the FIGO stage (*n* = 42; 55%), histological types (*n* = 38; 49%), and tumor size (*n* = 37; 48%) (Figure 2). There were at least six forms of lymph nodes included in the prognostic models. The version of FIGO stage used for patients was presented only in 19 (25%) models. Treatment was considered a predictor in 18 (23%) models.

The modeling method used most frequently in these models was Cox proportional hazard regression (*n* = 68; 88%), and machine learning was also used in five (6%) models. Model presentation was available for most prognostic models (*n* = 67; 87%), but only 16 (21%) models had a regression formula (Table 2).

**Table 2:** Methodological characteristics of the development of prognostic models for patients with cervical cancer by clinical stage.

**Methodological items**	**All models (77 models)**	**Early stage (29 models)**	**Locally advanced (27 models)**	**All stage (21 models)**	***P*-value**
Sample size, median (IQR)	549 (203.5–843)	330 (119–788)	314 (234–833)	1,501 (371–4,220)	0.011
Number of events, median (IQR)	77 (40.5–187)	47 (19.5–96.5)	106 (52.5–246.75)	166 (45.25–696.75)	0.005
Events per variable					
Not machine learning					0.006
EPV <10	35 (49)	19 (70)	11 (41)	5 (28)	
EPV 10–20	3 (4)	1 (4)	0 (0)	2 (11)	
EPV >20	22 (31)	2 (7)	12 (44)	8 (44)	
No information	12 (17)	5 (19)	4 (15)	3 (17)	
Machine learning					1.000
EPV <100	5 (100)	2 (100)	0 (0)	3 (100)	
EPV ≥100	0 (0)	0 (0)	0 (0)	0 (0)	
Internal validation					<0.001
Bootstrapping	35 (45)	10 (34)	20 (74)	5 (24)	
Cross-validation	9 (12)	2 (7)	3 (11)	4 (19)	
Random split	6 (8)	0 (0)	1 (4)	5 (24)	
Resampling	1 (1)	1 (3)	0 (0)	0 (0)	
Not reported	1 (1)	1 (3)	0 (0)	0 (0)	
No internal validation	25 (32)	15(52)	3 (11)	7 (33)	
Modeling method					0.113
Cox hazard model	68 (88)	26 (90)	26 (96)	16 (76)	
Logistic regression	2 (3)	1 (3)	1 (4)	0 (0)	
Machine learning	5 (6)	2 (7)	0 (0)	3 (14)	
Discriminant analysis	1 (1)	0 (0)	0 (0)	1 (5)	
Not reported	1 (1)	0 (0)	0 (0)	1 (5)	
Missing data handling					0.092
Multiple imputation	4 (5)	1 (3)	3 (11)	0 (0)	
Complete case analysis	31 (40)	9 (31)	9 (33)	13 (62)	
No information	42 (55)	19 (66)	15 (66)	8 (38)	
Model presentation					<0.001
Full equation	9 (12)	8 (28)	0 (0)	1 (5)	
Nomogram	46 (60)	10 (34)	25 (93)	11 (52)	
Sum score	4 (5)	2 (7)	0 (0)	2 (10)	
CART	1 (1)	1 (3)	0 (0)	0 (0)	
More than one method	7 (9)	3 (10)	2 (7)	2 (10)	
None	10 (13)	5 (17)	0 (0)	5 (24)	
Discrimination					0.044
C-index or AUROC	68 (88)	24 (83)	27 (100)	17 (81)	
None	9 (12)	5 (17)	0 (0)	4 (19)	
Calibration					0.087
Calibration plot	45 (58)	13 (45)	20 (74)	12 (57)	
None	32 (42)	16 (55)	7 (26)	9 (43)	

*Values are numbers (percentages^a^) unless stated otherwise*.

a *Some percentages do not add up to 100%, owing to rounding off*.

*IQR, interquartile range; EPV, events per variable; CART, Classification and Regression Tree; C-index, concordance index; AUROC, area under the receiver operating characteristic*.

### Prognostic Models for Patients With Early-Stage Cervical Cancer

Twenty-nine models were developed for patients with early-stage cervical cancer. The sample size (*p* = 0.011), number of events (*p* = 0.005), and events per variable in models that were not based on machine learning (*p* = 0.006) were significantly smaller in models for patients with early-stage cervical cancer. The predictors used most frequently in these models were lymph nodes status (*n* = 22; 76%), lymph vascular space invasion (LVSI) (*n* = 14; 48%), depth of stromal invasion (DOI) (*n* = 13, 45%), FIGO stage (*n* = 12; 41%), histological types (*n* = 11; 38%), tumor size (*n* = 11; 38%), and parametrium invasion (*n* = 11; 38%). LVSI (*p* = 0.003) and DOI (*p* < 0.001) were used significantly more frequently in the models for patients with early-stage cervical cancer (Figure 2).

A C-index or an AUROC was reported for 24 (83%) models, and the remaining five (19%) did not report any discrimination metric. The discrimination estimates ranged from 0.565 to 0.959. Less than half of the models (*n* = 14; 48%) performed internal validation. The calibration plot was used in 13 (45%) models, and the remaining models performed no calibration assessment. The relatively recommended prognostic model for patients with early-stage cervical cancer was the Je et al. model (27).

### Prognostic Models for Patients With Locally Advanced Cervical Cancer

In terms of the 27 (35%) prognostic models developed for locally advanced cervical cancer, the predictors encountered most frequently also included lymph node status (*n* = 23; 85%), tumor size (*n* = 16; 59%), FIGO stage (*n* = 15; 56%), histological types (*n* = 13; 48%), and age (*n* = 10; 37%) (Figure 2). The differences of internal validation (*p* < 0.001), model presentation (*p* < 0.001), and discrimination evaluation (*p* = 0.044) were significant across three groups. Bootstrapping and nomogram were used more frequently in the models for patients with locally advanced cervical cancer compared to the others. A C-index or an AUROC was reported for 17 (81%) models, which was significantly higher than the other two types, and the discrimination estimates ranged from 0.62 to 0.86. Calibration was assessed properly for 20 (74%) models with a calibration plot. The Prediction Model of Failure in Locally Advanced Cervical Cancer (PREFACE) was the relatively recommended model (28).

### Prognostic Models for All-Stage Cervical Cancer Patients

There were 21 (27%) models developed for the prognosis of all-stage cervical cancer patients. The predictors used commonly were FIGO stage (*n* = 15; 71%), histological type (*n* = 14, 67%), age (*n* = 13; 62%), lymph nodes status (*n* = 12; 57%), grade (*n* = 11, 52%), and tumor size (*n* = 10; 48%). Grade (*p* = 0.014) and race (*p* < 0.001) were used significantly more commonly in the models for all-stage cervical cancer patients (Figure 2). A C-index or an AUROC was reported for 17 (81%) models. The discrimination estimates ranged from 0.616 to 0.897. Two-thirds of the models (*n* = 14; 67%) had internal validation. More than a half of the (*n* = 12; 57%) models were assessed with a calibration plot. The Wang et al. model was relatively recommended (29).

### External Validation Studies

Of all models included, 21 (27%) were externally validated once, and three (4%) were validated twice. The median sample size and the median number of events were 211 (101–653) and 39 (18.5–83.5), respectively. Two (7%) external validation studies did not report any discrimination metric, and that of the remaining 25 studies ranged from 0.52 to 0.88. Fifteen (56%) models were assessed with calibration. The prognostic models that were externally validated twice were the SNU/AM (30), KROG 12-08 (8), and PREFACE (28), and the PREFACE model had better performance, with good calibration and a C-index of 0.67. Supplementary Material 4 presents detailed information on all the prognostic models.

### Risk of Bias of the Included Studies

**Figure 3:**
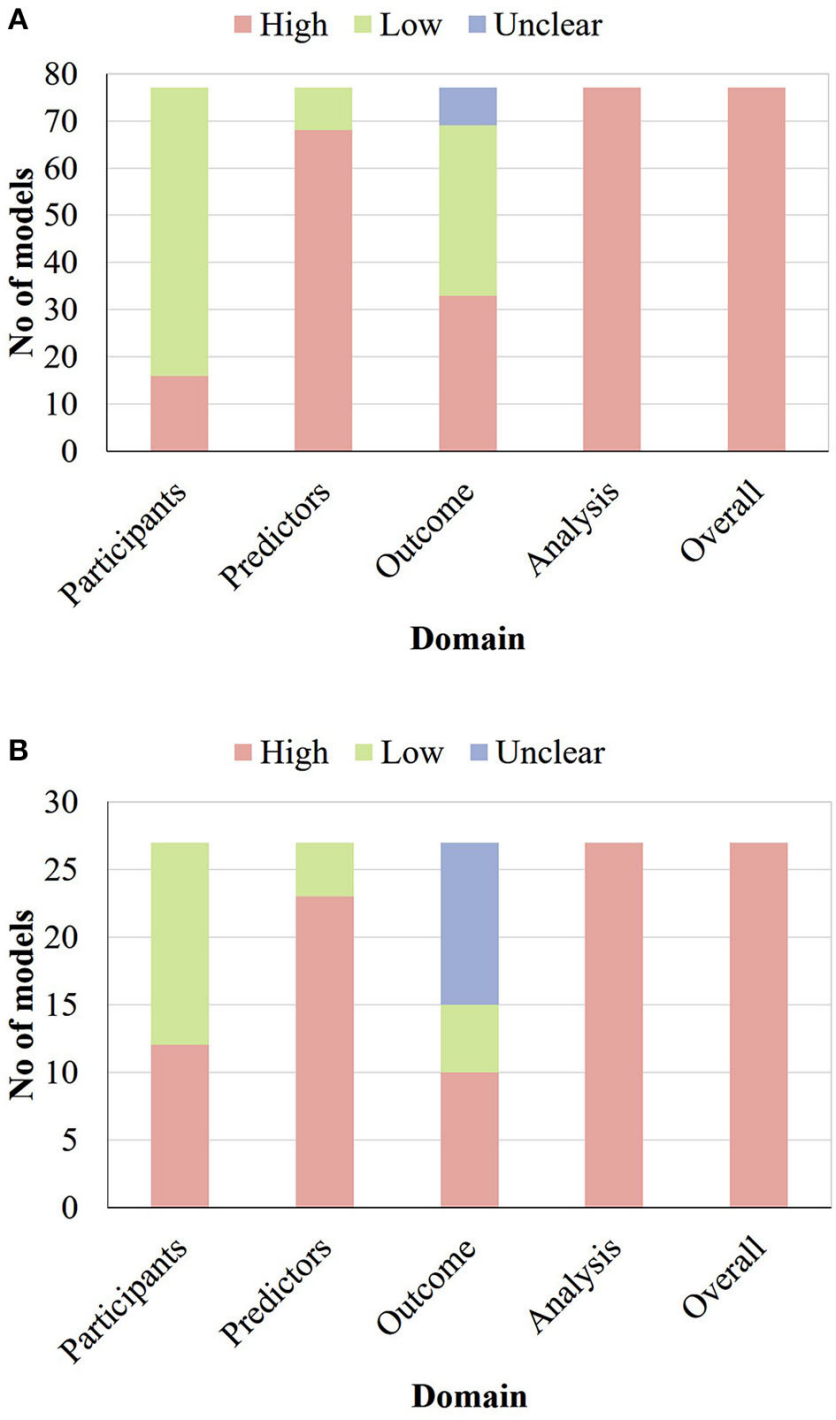
Risk of bias assessment (using PROBAST) based on four domains across 77 prognostic model development studies related to cervical cancer **(A)** and across 27 external validation efforts of prognostic models related to cervical cancer **(B)**.

The PROBAST was used to assess the risk of bias of all studies developing or externally validating prognostic models. None of the models developed or validated were assessed as being at low ROB; the summary by domain is shown in Figure 3. As for the model development studies, 61 (79%), nine (11%), and 33 (47%) models were at low ROB for participants, predictors, and outcome, respectively, but none of the models were at low ROB for analysis. Similarly, among 27 external validation efforts, 15 (56%), four (15%), and five (19%) models were at low ROB for participants, predictors, and outcome, respectively, but none of the models were at low ROB for analysis. Specific signal questions and the result of the assessment are in Supplementary Material 5. The full risk of bias assessment table can be found in Supplementary Material 6.

The main problems with regards to the analysis domain included the inadequate number of events (68% for development and 93% for validation), the incomplete evaluation of model performance (64% for development and 74% for validation), the improper handling of missing data (86% for development and 89% for validation), and the incomplete procedure of internal validation (97% for development).

## Discussion

Our systematic review revealed the detailed characteristics of 77 prognostic models for the prediction of clinical outcomes in cervical cancer patients, but none of the models developed or validated were assessed as being at low ROB with PROBAST mainly because of the limitations in modeling methodology and model presentation. Relatively, the PREFACE model predicting 5-year distant recurrence in locally advanced cervical cancer patients appears to be the most reliable model.

In terms of the geographical distribution of the research population, most of the models were developed and externally validated in countries with high and very high human development index (HDI), especially in the United States, China, and Korea where the incidence of cervical cancer is relatively high rather than in less developed countries like Africa or Melanesia where the incidence and mortality were the highest (2). Prognostic models tailored to less developed countries and external validation are needed before the generalization and application. In addition, from the perspective of predictor choice, the most commonly used predictors in all eligible prognostic models were different types of tumor characteristics, which were also mentioned in the NCCN Clinical Practice Guidelines (31). The LVSI and DOI were more frequently used as predictors in the models for patients with early-stage cervical cancer. The recommended treatment for patients with early-stage cervical cancer is surgery, and these predictive factors can be obtained with more accurate results in postoperative pathological testing (31). Grade and race were also more frequently used as predictors in models for all-stage cervical cancer patients, mainly because of the generalization of these predictors for all cervical cancer patients. Our research suggested that some issues related to predictors should be noted when developing, validating, or using prognostic models. First, the forms of predictors included in prognostic models are not consistent. For example, at least six forms of lymph node status were involved in existing models (29). A practical problem is that the effect of each form may be different, and which one is superior requires research to examine, which can help subsequent studies choose the appropriate one. Second, the FIGO stage is also crucial to the progression of cervical cancer, but the staging system has been revised at least three times during the past decades (32, 33). Researchers should confirm the version of FIGO stage they used ahead of model development or application. Third, the accuracy of measurement and the availability of predictors should be also considered. The tumor characteristics mentioned above are usually measured by different imaging evaluation and pathologic examination, which is subjective and may have inadequate accuracy (34). Therefore, parallel assessment and blindness are necessary during measurement procedures to ensure quality. Additionally, despite the increasing number of research on the predictive value of omics (35–37), the inclusion of omics into a conventional prediction model needs to be discussed considering the accessibility of these predictors and cost-effectiveness issues, especially in countries with middle or low income. Besides this, treatment is very important for the prognosis of cervical cancer patients, and different surgical methods may lead to different survival outcomes (38), but treatments were considered a predictor only in 18 models, which indicates that prognostic models tailored to different treatment modalities are needed.

Our systematic review showed several methodological pitfalls in the development or validation of the models, especially in the analysis domain which was the main cause of high ROB. Similar to reviews assessing the quality of prognostic models for other diseases including oropharyngeal cancer, chronic lymphocytic leukemia, and chronic obstructive pulmonary disease (19, 20, 39, 40), inadequate sample size, improper handling of missing data, and incomplete evaluation of model performance were the main problems in analysis domain and needed to be emphasized. Besides this, several other methodological details should also be noted. First, some common problems of internal validation exist, and the proportion of external validation was low. Only a half of the studies applied internal validation properly by using bootstrapping or cross-validation techniques to overcome overfitting; however, almost none of the bootstrapping or cross-validation techniques used in these studies were applied in all model development procedures such as variable selection, which was also consistent with other studies (41). It is one of the most overlooked technical aspects at present, but it violates the guidance and recommendations of PROBAST on internal validation (25, 42), indicating that the recommendation needs to be further strengthened. Additionally, only one third of the models have been externally validated, although the demonstration of the performance of a model in an independent population is a necessary step before recommending its widespread use (43). Researchers generally do not have access to multiple data, and external validation might be limited because of that. Data sharing can offer the possibility of making the most of all available data, which should be promoted in the future (44). Second, besides the most common Cox proportional hazard regression that was suitable for survival data, machine learning was also adopted for prognostic prediction in cervical cancer from 2019 (45). The discrimination parameter of the machine learning model was better than the traditional one (0.795 vs. 0.784) (45). The model performance could therefore be partially improved, but researchers should also attend to the difficulty of interpretation of algorithm and requirement of large sample size (46). In addition, it should be noted that different predicted outcomes have corresponding suitable modeling methods, and the same type of outcome also has alternative statistical models; therefore, researchers should consider the characteristics of different modeling methods when choosing one (47). Third, the model presentation was also inappropriate, with almost one-seventh of the models having no presentation and only one-fifth providing the full model equation. Researchers should provide not only concise and proper presentation formats but also the full model equation to enable independent external validation, update, and recalibration (48). Compared with the above-mentioned two issues, this one is much easier to realize. Both authors and journals should pay attention to it to directly facilitate the validation and generalization of prognostic models.

In terms of the differences of methodological details across the three groups, the problem of adequate sample size was most severe in models for patients with early-stage cervical cancer. Early-stage patients have a better prognosis, leading to a fewer number of events. The methodological details (including internal validation, model presentation, and discrimination evaluation) of models for patients with locally advanced cervical cancer were generally better than the other two categories. We think that the reason for those differences might not be relevant to the features of patients in different stages. Indeed the all-stage cervical cancer patients included both patients with early-stage cancer and patients with locally advanced cancer. We think that the choice of method should be mainly related to the researchers. We added new analysis to compare the year of publication and the involvement of methodological experts across the three groups. We found that the publication years of the models were similar across the three groups, and the proportion of models involving methodological experts was not high in the locally advanced group (Supplementary Material 3). Although the current clues cannot easily explain the above-mentioned phenomenon, this result indicated that a couple of research teams did better at methodological details, and it also takes lead to improve the qualities of models in this field.

Although all of the existing models have high ROB, current research still inevitably needs the guidance of prognostic models on clinical intervention, therapeutic strategy, clinical trial, and so on. Clinicians need to choose the most appropriate and corresponding models to predict the prognosis of patients at different stages. Appropriate models should possess high quality, good model performance, strict validation, and available predictors. Before the development of new models with better quality, the Je et al. model (27), PREFACE model (28), and Wang et al. model (29) were relatively recommended for early stage, locally advanced, and all-stage cervical cancer patients, respectively. The Je et al. (27) model was recommended based on the relatively standardized measurement of predictors and outcome, large sample size, complete evaluation and good result of model performance, and internal validation. The PREFACE model was recommended based on the barely enough sample size and external validation in both Korea and Spain with acceptable performance. The Wang et al. (29) model was based on a large database with good data quality and enough sample size and was externally validated in an independent population with good performance. Of course, researchers should notice the high ROB and existing problems of these models when using them. The serum squamous cell carcinoma antigen, a predictor included in the PREFACE model, can only predict the prognosis of cervical squamous cell carcinoma (49, 50). The Je et al. (27) model still needs to be externally validated, and some analysis details including missing data were handled improperly in the Wang et al. (27) model. Therefore, external validation and more high-quality prognostic models tailored to different cervical cancer patients are needed before models can be implemented in clinical practice.

To get prognostic models with good predictive capability for clinical practice, researchers should pay attention to each detail in the modeling process. The larger value of discrimination and calibration does not always mean a better model; instead it is also important to assess the risk of bias of the model from multiple domains. Although these domains are very detailed, it is still recommended to pay attention and value to avoid misuse. On the basis of the aforementioned problems, besides some common issues including recruiting enough participants to obtain enough outcome events, applying imputation techniques to handle missing data, and providing complete model performance measures, the following recommendations could be stated to improve the research on prognostic models for cervical cancer. First, model development studies should apply internal validation to adjust for overfitting and include all model development procedures. Second, several modeling guidelines of methodology and reporting specific to machine learning have been developed recently (51, 52), and researchers should follow these guidelines to correctly apply machine learning for prediction in the future. Third, researchers should carefully consider the intended users, settings, and timing when choosing the format to present the model, and a final prognostic model equation should always be presented. Finally, research is mostly concentrated in countries with high and very high HDI now; similar studies in other populations are needed.

To our knowledge, this is the first systematic review to provide a comprehensive overview of available studies on prognostic models for different outcomes of cervical cancer according to the CHARMS and PROBAST tools. Nevertheless, it has some limitations. First, we only included studies published in English and did not search gray literature, but the missing models due to this are limited in usage and usually of relatively low quality. Second, a quantitative analysis was not conducted due to the heterogeneity of participants and outcomes.

## Conclusion

The number of prognostic models for cervical cancer has increased steadily in the past decade, tripling in annual growth in 2019, and the performance of these models varies. However, all of them are at high risk of bias mainly because of unsatisfactory statistical analysis. High-quality prognostic models tailored to different stages of cervical cancer, external validation by an independent population, and head-to-head comparisons of existing models are needed to inform clinical practice better.

## Data Availability Statement

The original contributions presented in the study are included in the article/Supplementary Material, further inquiries can be directed to the corresponding author/s.

## Author Contributions

BH, WC, LL, and SW designed the study. BH, WC, HZ, HC, and YZ did the literature search and the data extraction. BH and WC performed the initial analysis of data and wrote the first draft of the manuscript. ZH, SW, and SZ contributing to the interpretation of data. All the authors contributed to the critical revision of the manuscript for important intellectual content and approved the final version.

## Conflict of Interest

The authors declare that the research was conducted in the absence of any commercial or financial relationships that could be construed as a potential conflict of interest.
