# Supplementary material for: Prediction Models for Prognosis of Cervical Cancer: Systematic Review and Critical Appraisal
Source: Front Public Health. 2021 May 7;9:654454. doi: 10.3389/fpubh.2021.654454 (PMC8137851; doi:10.3389/fpubh.2021.654454)
Supplement: Supplementary file 1 [file Table_1.DOCX]

**Supplement file 2**

Search strategy:

PubMed

(predict*[title/abstract] or progn*[title/abstract] or “risk score”[title/abstract] or “risk calculation”[title/abstract] or “risk assessment”[title/abstract] or “c statistic”[title/abstract] or discrimination[title/abstract] or calibration[title/abstract] or auc[title/abstract] or “area under the curve”[title/abstract] or “area under the receiver operator characteristic curve”[title/abstract]) and ("uterine cervical neoplasms"[mesh] or ((cervical[title/abstract] or cervix[title/abstract]) and (cancer*[title/abstract] or carcinoma*[title/abstract] or neoplasm*[title/abstract] or tumor*[title/abstract])))

Embase

(predict*:ab,ti OR progn*:ab,ti OR 'risk score':ab,ti OR 'risk calculation':ab,ti OR 'risk assessment':ab,ti OR 'c statistic':ab,ti OR discrimination:ab,ti OR calibration:ab,ti OR auc:ab,ti OR 'area under the curve':ab,ti OR 'area under the receiver operator characteristic curve':ab,ti) AND ('uterine cervix cancer'/exp OR ((cervical:ab,ti OR cervix:ab,ti) AND (cancer*:ab,ti OR carcinoma*:ab,ti OR neoplasm*:ab,ti OR tumor*:ab,ti)))

Cochrane (review, protocol, trial)

#1 (predict* OR progn* OR “risk prediction” OR “risk score” OR “risk calculation” OR “risk assessment” OR “c statistic” OR discrimination OR calibration OR AUC OR “area under the curve” OR “area under the receiver operator characteristic curve”):ti,ab,kw

#2 MeSH descriptor: [Uterine Cervical Neoplasms] explode all trees

#3 ((cervical OR cervix) AND (cancer* OR carcinoma* OR neoplasm* OR tumor*)):ti,ab,kw

#4 #1 AND (#2 OR #3)
