# Supplementary material for: Prediction Models for Prognosis of Cervical Cancer: Systematic Review and Critical Appraisal
Source: Front Public Health. 2021 May 7;9:654454. doi: 10.3389/fpubh.2021.654454 (PMC8137851; doi:10.3389/fpubh.2021.654454)
Supplement: Supplementary file 2 [file Table_2.DOCX]

**Supplemental file 5**

Signal questions of PROBAST:

1.1 Were appropriate data sources used, e.g., cohort, randomized controlled trial, or nested case–control study data?

1.2 Were all inclusions and exclusions of participants appropriate?

2.1 Were predictors defined and assessed in a similar way for all participants?

2.2 Were predictor assessments made without knowledge of outcome data?

2.3 Are all predictors available at the time the model is intended to be used?

3.1 Was the outcome determined appropriately?

3.2 Was a prespecified or standard outcome definition used?

3.3 Were predictors excluded from the outcome definition?

3.4 Was the outcome defined and determined in a similar way for all participants?

3.5 Was the outcome determined without knowledge of predictor information?

3.6 Was the time interval between predictor assessment and outcome determination appropriate?

4.1 Were there a reasonable number of participants with the outcome?

4.2 Were continuous and categorical predictors handled appropriately?

4.3 Were all enrolled participants included in the analysis?

4.4 Were participants with missing data handled appropriately?

4.5 Was selection of predictors based on univariable analysis avoided? (Model development studies only)

4.6 Were complexities in the data (e.g., censoring, competing risks, sampling of control participants) accounted for appropriately?

4.7 Were relevant model performance measures evaluated appropriately?

4.8 Were model overfitting and optimism in model performance accounted for? (Model development studies only)

4.9 Do predictors and their assigned weights in the final model correspond to the results from the reported multivariable analysis? (Model development studies only)

A


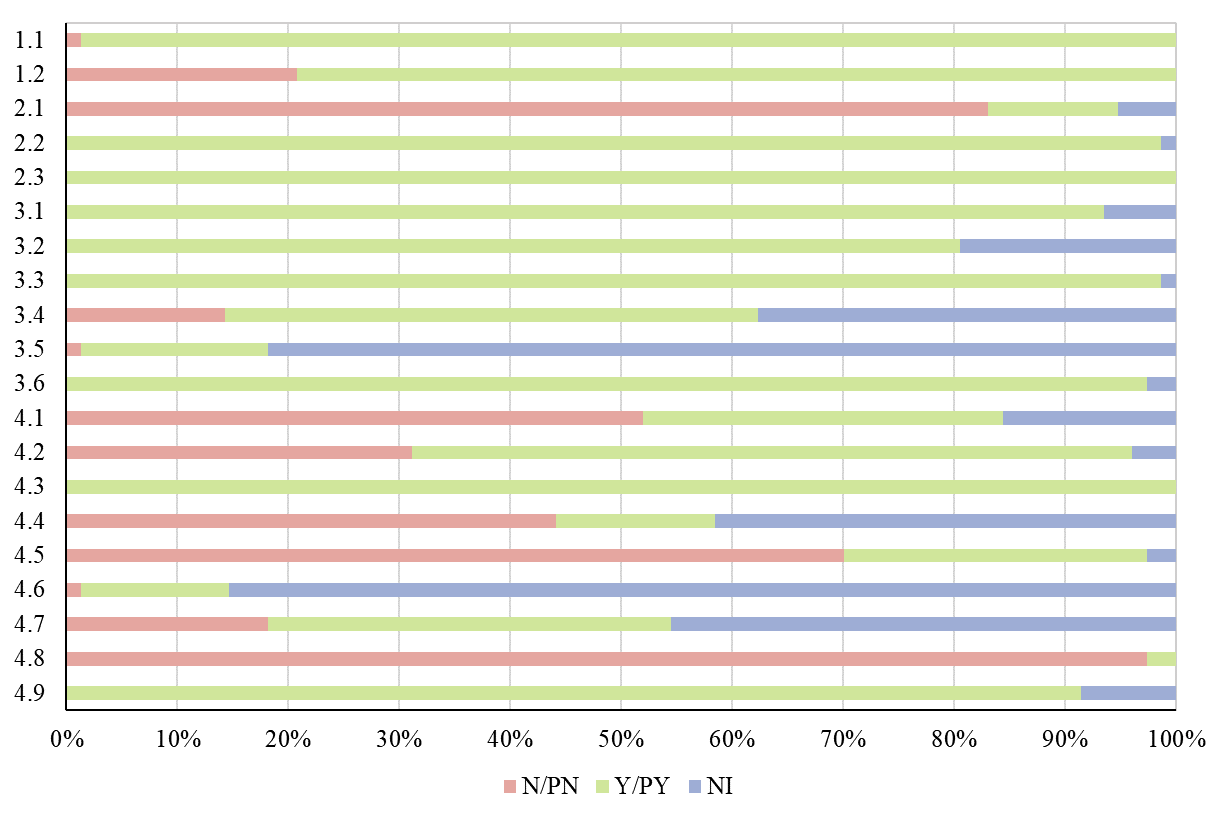


B


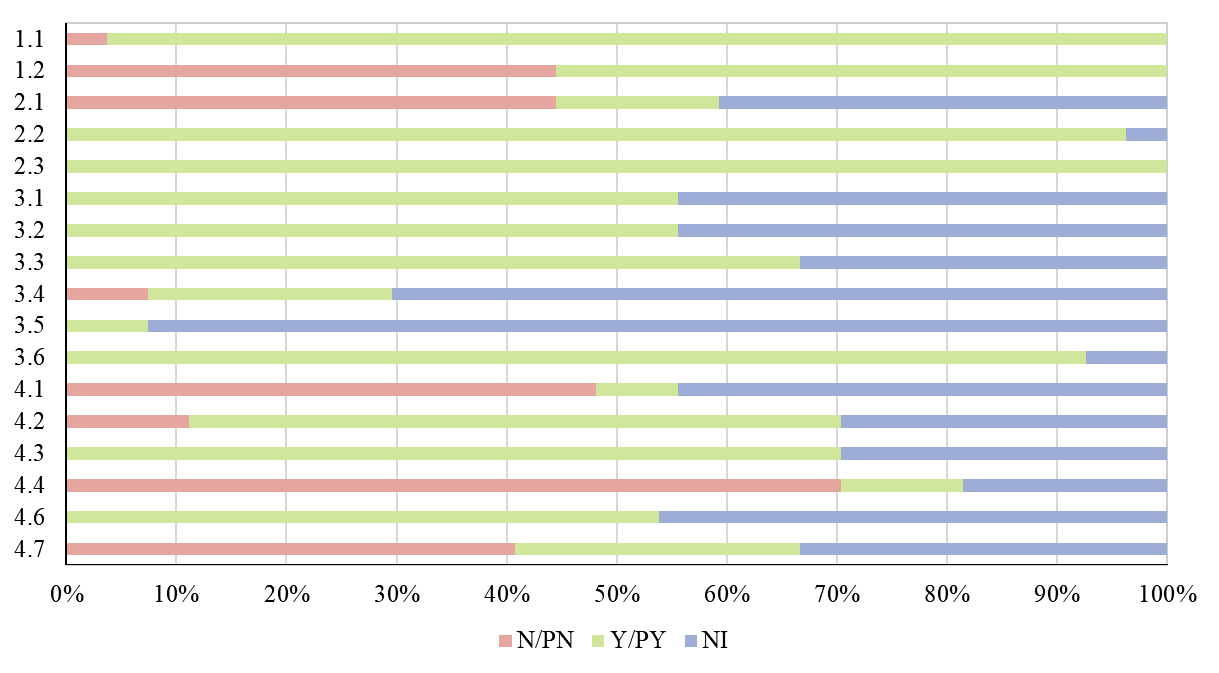


**Figure S5** The result of 20 signal questions of model development studies (A) and the result of 17 applicable signal questions of external validation studies (B)

N=no; PN=probably no; Y=yes; PY=probably yes; NI=no information.
