# Supplementary material for: Prediction Models for Prognosis of Cervical Cancer: Systematic Review and Critical Appraisal
Source: Front Public Health. 2021 May 7;9:654454. doi: 10.3389/fpubh.2021.654454 (PMC8137851; doi:10.3389/fpubh.2021.654454)
Supplement: Supplementary file 4 [file Table_4.DOCX]

**Supplement file 3**

**Prognostic models for patients with early-stage cervical cancer:**

**Table S2.A** Prognostic models for patients with early-stage cervical cancer

| **No.** | **Author (year)** | **Title** | **The FIGO stage of Participants** |
| --- | --- | --- | --- |
| 1 | Lai (1999) | Preoperative Prognostic Variables and the Impact of Postoperative Adjuvant Therapy on the Outcomes of **Stage IB or II** Cervical Carcinoma Patients with or without Pelvic Lymph Node Metastases: An Analysis of 891 Cases | IB or II |
| 2 | Pieterse (2008) | An individual prediction of the future (disease-free) survival of patients with a history of **early-stage** cervical cancer, multistate model | I–IIA |
| 3 | Kim (2010) | Postoperative nomogram predicting risk of recurrence after radical hysterectomy for **early-stage** cervical cancer | I–IIA |
| 4 | Biewenga (2011) | Prognostic model for survival in patients with **early stage** cervical cancer | IA2-IIA |
| 5 | Khunamornpong (2013) | Prognostic model in patients with **early-stage squamous cell carcinoma** of the uterine cervix: a combination of invasive margin pathological characteristics and lymphovascular space invasion | IB1-2 to IIA |
| 6 | Kusy (2013) | Application of gene expression programming and neural networks to predict adverse events of **radical hysterectomy** in cervical cancer patients | IA2 to IIB, treated surgically |
| 7 | Lee (2013) | Individualized Prediction of Overall Survival After Postoperative Radiation Therapy in Patients With **Early-Stage** Cervical Cancer: A Korean Radiation Oncology Group Study | IB-IIA |
| 8 | Je (2014) | A nomogram predicting the risks of distant metastasis following **postoperative radiotherapy** for uterine cervical carcinoma: a Korean radiation oncology group study (KROG 12-08) | I-IIA |
| 9 | Yu (2014) | Prediction of **local recurrence** in cervical cancer by a Cox model comprised of lymph node status, lymph-vascular space invasion, and intratumoral Th17 cell-infiltration | II, underwent primary surgery |
| 10 | Zhou (2015) | Establishing a Nomogram for **Stage IA-IIB Cervical Cancer** Patients after Complete Resection | IA-IIB, treated with radical surgery |
| 11 | Cao (2016) | Upregulation of long noncoding RNA SPRY4-IT1 correlates with tumor progression and poor prognosis in cervical cancer | IB-IIB, underwent radical resections |
| 12 | Lee (2017) | A postoperative scoring system for distant recurrence in node-positive cervical cancer patients after **radical hysterectomy** and pelvic lymph node dissection with para-aortic lymph node sampling or dissection | IA2 to IIB, early-stage |
| 13 | Obrzut (2017) | Prediction of 5-year overall survival in cervical cancer patients treated with **radical hysterectomy** using computational intelligence methods | IA2-IIB |
| 14-16 | Yoon (2017) | Validation of Nomograms for Survival and Metastases after **Hysterectomy** and Adjuvant Therapy in Uterine Cervical Cancer with Risk Factors | I-IIA |
| 17 | Zheng (2017) | Nomogram predicting overall survival in **operable** cervical cancer patients | IA1-IIA，underwent operations for early stage |
| 18 | Je (2018) | Risk prediction model for disease-free survival in women with **early-stage** cervical cancers following postoperative (Chemo)radiotherapy | I or IIA |
| 19 | Gülseren (2019) | Postoperative nomogram for the prediction of disease-free survival in lymph node-negative stage I–IIA cervical cancer patients **treated with radical hysterectomy** | IA2–IIA |
| 20 | Hong (2019) | Enhancing prognosis prediction using pre-treatment nodal SUVmax and HPV status in cervical squamous cell carcinoma | IA3–IIA |
| 21 | Huang (2019) | Prognostic Value of Preoperative Systemic Immune-Inflammation Index in Patients with Cervical Cancer | IA–IIA, receiving radical resection |
| 22 | Obrzut (2019) | Prediction of 10-year Overall Survival in Patients with **Operable** Cervical Cancer using a Probabilistic Neural Network | IA2–IIB |
| 23-26 | Paik (2019) | Prognostic Model for Survival and Recurrence in Patients with **Early-Stage** Cervical Cancer: A Korean Gynecologic Oncology Group Study (KGOG 1028) | IB-IIA |
| 27 | Zuo (2019) | Nomograms based on HPV load for predicting survival in cervical squamous cell carcinoma: An observational study with a long-term follow-up | IB1-IIA |
| 28 | Huang (2020) | Prognostic Factors and Local Treatment Modalities of Small-Cell Carcinoma of the Cervix: An Analysis According to the International Federation of Gynecology and Obstetrics Stage | I-IIA |
| 29 | Chao (2020) | A Novel Prognostic Marker Systemic Inflammation Response Index (SIRI) for Operable Cervical Cancer Patients | IA–IIA |

**Prognostic models for patients with locally advanced cervical cancer:**

**Table S2.B** Prognostic models for patients with locally advanced cervical cancer

| **No.** | **Author (year)** | **Title** | **The FIGO stage of Participants** |
| --- | --- | --- | --- |
| 1 | Tseng (2010) | Prognostic nomogram for overall survival in **stage IIB-IVA** cervical cancer patients treated with **concurrent chemoradiotherapy** | IIB-IVA |
| 2 | Seo (2011) | Nomogram prediction of overall survival after **curative irradiation** for uterine cervical cancer | II, III, or IVA |
| 3 | Kang (2012) | Risk assessment tool for distant recurrence after platinum-based concurrent chemoradiation in patients with **locally advanced cervical cancer**: a Korean gynecologic oncology group study | IIB to IVA or IB2 to IIA bulky |
| 4-6 | Kidd (2012) | FDG-PET-based prognostic nomograms for **locally advanced cervical cancer** | IB1- IVA |
| 7 | Shim (2012) | Risk assessment model for overall survival in patients with **locally advanced cervical cancer** treated with definitive concurrent chemoradiotherapy | IB2-IVA |
| 8-10 | Rose (2015) | Nomograms Predicting Progression-Free Survival, Overall Survival, and Pelvic Recurrence in **Locally Advanced Cervical Cancer** Developed From an Analysis of Identifiable Prognostic Factors in Patients From NRG Oncology/Gynecologic Oncology Group Randomized Trials of Chemoradiotherapy | IB2-IVA |
| 11 | Huang (2016) | Prognostic risk model development and prospective validation among patients with cervical cancer stage IB2 to IIB submitted to neoadjuvant chemotherapy | IB2 to IIB |
| 12-13 | Marchetti (2018) | Survival nomograms after curative neoadjuvant chemotherapy and radical surgery for stage IB2-IIIB cervical cancer | IB2-IIIB |
| 14 | Gao (2019) | Multiparametric PET/MR (PET and MR-IVIM) for the evaluation of early treatment response and prediction of tumor recurrence in patients with **locally advanced cervical cancer** | IIb to IVA |
| 15-18 | Wang (2019) | Nomograms predicting survival and patterns of failure in patients with cervical cancer treated with **concurrent chemoradiotherapy**: A special focus on lymph nodes metastases | IB1-IVA |
| 19 | Zuo (2019) | Nomograms based on HPV load for predicting survival in cervical squamous cell carcinoma: An observational study with a long-term follow-up | IIB-IVA |
| 20 | Zhang (2019) | Score for the Overall Survival Probability of Patients With First-Diagnosed Distantly Metastatic Cervical Cancer: A Novel Nomogram-Based Risk Assessment System | Metastatic cervical cancer |
| 21-22 | Yang (2020) | Prognostic Nomograms Predicting Survival in Patients With Locally Advanced Cervical Squamous Cell Carcinoma: The First Nomogram Compared With Revised FIGO 2018 Staging System | IB–IVA, LACC |
| 23 | Huang (2020) | Prognostic Factors and Local Treatment Modalities of Small-Cell Carcinoma of the Cervix: An Analysis According to the International Federation of Gynecology and Obstetrics Stage | IIB-IV |
| 24-25 | Lee (2020) | Prognosis-Predicting Model Based on [(18)F]fluorodeoxyglucose PET Metabolic Parameters in Locally Advanced Cervical Cancer Patients Treated with Concurrent Chemoradiotherapy: Multi-Center Retrospective Study | ≥IIB |
| 26-27 | Mu (2020) | 18F-FDG PET/CT Habitat Radiomics Predicts Outcome of Patients with Cervical Cancer Treated with Chemoradiotherapy | IIB-IVA |

**Prognostic models for all stage cervical cancer patients:**

**Table S2.C** Prognostic models for all stage cervical cancer patients

| **No.** | **Author (year)** | **Title** | **The FIGO stage of Participants** |
| --- | --- | --- | --- |
| 1 | Te Velde (1987) | Prognostic Significance of Pretreatment Variables in Patients with Invasive Cervical Cancer | I-III, macro-invasive patients |
| 2 | Ng (1992) | A scoring system for predicting recurrence of cervical cancer | IB-IIIA |
| 3 | Polterauer (2012) | Nomogram prediction for overall survival of patients diagnosed with cervical cancer | I-IV, all consecutive patients, |
| 4 | Huang (2015) | Chemotherapy-induced neutropenia during adjuvant treatment for cervical cancer patients: development and validation of a prediction model | Patients with chemotherapy |
| 5 | Sala (2015) | Complementary Prognostic Value of Pelvic Magnetic Resonance Imaging and Whole-Body Fluorodeoxyglucose Positron Emission Tomography/Computed Tomography in the Pretreatment Assessment of Patients With Cervical Cancer | I-IV, all cervical cancer patients |
| 6-7 | Li (2016) | Development and validation of a surgical-pathologic staging and scoring system for cervical cancer | IA-III |
| 8 | Wang (2018) | A Prognostic Nomogram for Cervical Cancer after Surgery from SEER Database | I-IV, all cervical cancer patients |
| 9-10 | Matsuo (2019) | Survival outcome prediction in cervical cancer: Cox models vs deep-learning model | I-IV, invasive cervical cancer |
| 11-12 | Yang (2019) | Nomograms for predicting the survival rate for cervical cancer patients who undergo radiation therapy: a SEER analysis | I-IV, all cervical cancer patients |
| 13 | Qi (2020) | An Innovative Immune Score-Based Prognostic Nomogram for Patients with Cervical Cancer | I-IV |
| 14 | Xie (2020) | Calculating the overall survival probability in patients with cervical cancer: a nomogram and decision curve analysisbased study | I-IV |
| 15 | Li (2020) | A prognostic nomogram integrating novel biomarkers identified by machine learning for cervical squamous cell carcinoma | I-IV |
| 16 | Li (2020) | Moving beyond the Cox proportional hazards model in survival data analysis: a cervical cancer study | I-III |
| 17 | Liang (2020) | A prognostic model guides surgical resection in cervical squamous cell carcinoma | I-IV |
| 18-19 | Liu (2020) | Development and validation of a SEER-based prognostic nomogram for cervical cancer patients below the age of 45 years | I-IV |
| 20 | Xie (2020) | Characteristics and Treatments of Patients Aged 65 Years or Over with Cervical Cancer | I-IV |
| 21 | Yang (2020) | Dynamic prediction and prognostic analysis of patients with cervical cancer: a landmarking analysis approach | I-IV |

**Publication year (median (interquartile range)):**

Early-stage: 2017 (2014, 2019)

Locally advanced: 2019 (2012, 2020)

All stage: 2019 (2016, 2020)

**Proportion of models with methodological experts in the research team:**

Early-stage: 24%

Locally advanced: 22%

All stage: 59%
